# Preparation of a Red−Emitting, Chitosan−Stabilized Copper Nanocluster Composite and Its Application as a Hydrogen Peroxide Detection Probe in the Analysis of Water Samples

**DOI:** 10.3390/bios13030361

**Published:** 2023-03-09

**Authors:** Jiaojiao Lu, Dawei Wang, Xin Li, Wei Guo, Chunyuan Tian, Feng Luan, Xuming Zhuang

**Affiliations:** 1 College of Chemistry and Chemical Engineering, Yantai University, Yantai 264005, China; 2 Shandong Dyne Marine Biopharmaceutical Co., Ltd., Weihai 264300, China

**Keywords:** copper nanoclusters, chitosan, aggregation-induced emission, fluorescent probe, hydrogen peroxide

## Abstract

Hydrogen peroxide (H_2_O_2_) is an important reactive oxygen species that mediates a variety of physiological functions in biological processes, and it is an essential mediator in food, pharmaceutical, and environmental analysis. However, H_2_O_2_ can be dangerous and toxic at certain concentrations. It is crucial to detect the concentration of H_2_O_2_ in the environment for human health and environmental protection. Herein, we prepared the red-emitting copper nanoclusters (Cu NCs) by a one-step method, with lipoic acid (LA) and sodium borohydride as protective ligands and reducing agents, respectively, moreover, adding chitosan (CS) to wrap LA−Cu NCs. The as-prepared LA−Cu NCs@CS have stronger fluorescence than LA−Cu NCs. We found that the presence of H_2_O_2_ causes the fluorescence of LA−Cu NCs@CS to be strongly quenched. Based on this, a fluorescent probe based on LA−Cu NCs@CS was constructed for the detection of H_2_O_2_ with a limit of detection of 47 nM. The results from this research not only illustrate that the as--developed fluorescent probe exhibits good selectivity and high sensitivity to H_2_O_2_ in environmental water samples but also propose a novel strategy to prepare red-emitting copper nanoclusters (Cu NCs) by a one-step method.

## 1. Introduction

Copper nanoclusters (Cu NCs), which have attracted much attention as functionalized green metal nanomaterials, have been widely studied and applied in the fields of fluorescent probes, biosensing, and cell imaging [1,2,3]. Different from traditional fluorescent materials (such as semiconductor quantum dots, organic dyes, and polymer microspheres), Cu NCs with ultrasmall size endow them with diversified functions, tunable fluorescence, low biological toxicity, and low cost [4,5,6] also prompt Cu NCs to possess broad application prospects. At present, Cu NCs can be synthesized by many generally adopted methods, such as the chemical reduction method, template method, and electrochemical method [7,8,9], and the prepared Cu NCs show fluorescence emission that ranges from blue to red light [10]. However, most Cu NCs show blue fluorescent emission with a short emission wavelength and a small Stokes shift. The penetrating ability of the sample is weak, and there is background signal interference, which partly damages biological samples and limits the applicability of Cu NCs as fluorescent probes for highly sensitive detection and biological analysis [11,12]. Therefore, the development of red fluorescent Cu NCs with stable performance and a large Stokes shift has always been the focus of researchers.

Chitosan (CS), a glycosaminoglycan, is a product of partial deacetylation of chitin, with a wide range of sources, and the main biomass sources are fungi, animal bones, and the shells of shrimps and crabs [13,14,15]. As a multifunctional polymer material, CS has various excellent properties, such as biocompatibility, biodegradability, antibacterial activity, and nontoxicity [16,17], and is regarded as a good applicable raw material in many fields, such as the food industry, pharmaceutical industry, cosmetics, and biotechnology [18,19,20]. A valuable feature of CS is that its chemical structure contains many intrinsic oxygen and nitrogen functional groups, which can be used as the starting point for covalent modification or chitosan chain cross-linking. However, the poor solubility limits the further application of CS, and many studies have modified CS to prepare derivatives to improve its solubility. At present, CS can be modified by adjusting the degree of deacetylation and viscosity, introducing hydrophilic groups, and changing the solvent pH (weakly acidic) [21,22,23]. In addition, CS can self-assemble and combine with nanomaterials such as graphene and metal nanomaterials [24,25] through covalent bonds and hydrogen bonds, which not only expands the application of CS but also provides functionalized properties.

**Scheme 1:**
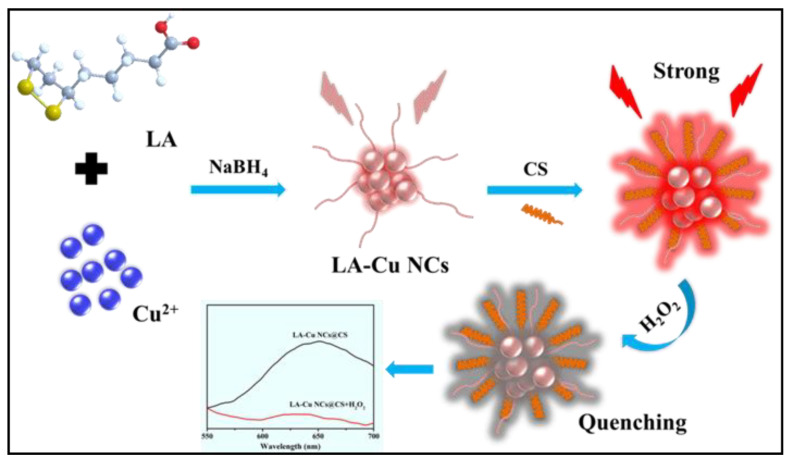
The preparation of the LA−Cu NCs@CS nanocomposite and the detection principle of H_2_O_2_.

The proposal of the aggregation-induced emission (AIE) effect fundamentally solved the problem of luminescence quenching caused by aggregation in solution, and the majority of researchers have performed much work on AIE [26,27,28]. The AIE enhancement effect is also an effective means for many metal nanoclusters to enhance fluorescence. In the aggregated state, intramolecular movement is restricted, which increases the coplanarity of the fluorescent molecules and contributes to fluorescence emission [29]. Several past studies [30,31,32] have shown that metal clusters can also produce AIE. Combined with these studies and our experimental results, we reasonably believe that the reason for the fluorescence of LA−Cu NCs@CS is related to the AIE effect of Cu NCs. After the coating of LA−Cu NCs with chitosan, the connection between Cu NCs and its ligand is closer, to produce AIE and emit strong red fluorescence. In this study, with lipoic acid (LA) as the ligand and sodium borohydride (NaBH_4_) as the reducing agent, LA−protected Cu NCs (LA−Cu NCs) were synthesized by a one-step method. The prepared LA−Cu NCs showed red fluorescence emission, but the stability was poor, the yield was low, and the NCs were difficult to separate and purify (Scheme 1). After adding CS, the dispersed LA−Cu NCs showed a state of aggregation, and the fluorescence intensity was increased by four times, showing AIE enhancement characteristics. Based on this, LA−Cu NCs@CS nanocomposites were prepared, and their related properties were explored. Finally, a fluorescent probe was constructed to realize the selective and sensitive detection of H_2_O_2_ in environmental water samples, and the limit of detection (LOD) was 47 nM in the detection range of 0.2–128 µM.

## 2. Materials and Methods

### 2.1. Materials

Lipoic acid (LA) and chitosan (CS) were purchased from Aladdin Reagent Co., Ltd. (Shanghai, China). Sodium borohydride (NaBH_4_) was obtained from Kelon Chemical Reagent Co., Ltd. (Chengdu, China). Copper nitrate trihydrate (Cu(NO_3_)_2_•3H_2_O), anhydrous ethanol, and acetic acid were purchased from Sinopharm Chemical Reagent Co., Ltd. (Shanghai, China). Hydrogen peroxide (H_2_O_2_) was purchased from Beilian Fine Chemicals Development Co., Ltd. (Tianjin, China). Different metal−ionic (Ag^+^, Al^3+^, Cd^2+^, Cr^2+^, Cu^2+^, Eu^3+^, Fe^2+^, Fe^3+^, Hg^2+^, Mn^2+^, Ni^2+^, Pb^2+^, and Zn^2+^) and anionic (F^−^ Cl^−^, Br^−^, I^−^, S^2−^, NO_2_^2−^, S_2_O_3_^2−^, SO_4_^2−^, Ac^−^, [Fe(CN)_6_]^3−^, CO_3_^2−^, and PO_4_^3−^) solutions were prepared by their respective crystal salts with the same concentration (32 µM). The tap water and lake water samples were randomly taken from the local tap water and Sanyuan Lake of Yantai University. All experimental water in this study was produced from a UPT−II−10T ultrapure water purifier (18.2 MΩ•cm). All chemical reagents were not further purified unless otherwise specified.

### 2.2. Apparatus

Scanning electron microscope (TEM) images were taken through a TSM-7900F electron microscope (JEOL Ltd., Tokyo, Japan). Transmission electron microscopy (TEM) and High resolution transmission electron microscope (HRTEM) images were taken through a JEM−2010 transmis XCsion electron microscope with an accelerating voltage of 200 kV (JEOL Ltd., Tokyo, Japan). Ultraviolet-visible absorption (UV-Vis) spectroscopy was obtained by a PerkinElmer Lambda 365 spectrometer (Shimfusa, Japan) with a wavelength interval of 5 nm. Fourier transform infrared (FT−IR) spectroscopy was performed using a Nicolet 5700 FT−IR spectrometer (Thermo Fisher Scientific, Waltham, MA, USA). X-ray photoelectron spectroscopy (XPS) was carried out using a Thermo ESCALAB−250 (Thermo Fisher Scient-ific, Waltham, MA, USA). The fluorescent spectrum was measured by an F−2700 spectrophotometer (Hitachi, Japan). Energy dispersive X-ray spectrometry (EDS) and EDS mapping elemental analysis were measured by an Ultim Extreme detector (Oxford Instruments Technology, Shanghai, China).

### 2.3. Synthesis of Nanocomposites

#### 2.3.1. Preparation of LA−Cu NCs

Seventy−two milligrams (0.35 mmol) of LA and 14 mg (0.37 mmol) of NaBH_4_ were accurately weighed into 16 mL ultrapure water and stirred thoroughly for 5 min at room temperature. Then, 700 µL of 25 mM Cu(NO_3_)_2_•3H_2_O was added to the above-mixed solution, and the color of the solution gradually changed from colorless to yellow-brown. After that, the dissolved oxygen in the mixed solution was pumped out with a vacuum pump, and then high-purity nitrogen was injected. The step was repeated three times to ensure that the oxygen was completely removed. Finally, the above-mixed solution was stirred and reacted for 6 h in an ice water bath (5–10 °C) to obtain a clear yellow-brown LA−Cu NC solution, which exhibited weak red fluorescence under 365 nm UV light. The prepared LA−Cu NC solution was stored at 4 °C for subsequent use.

#### 2.3.2. Preparation of LA−Cu NCs@CS

The operation was to add 1.6 mL 1 mg/mL CS−1% acetic acid solution (ratio of CuNCs and CS solutions = 10:1 *v/v*) to the preprepared LA−Cu NC solution, and there was a clear yellow precipitate in the solution. After vigorously stirring for 30 min at room temperature, a large amount of aggregated precipitate was generated, which emitted obvious red fluorescence under irradiation with 365 nm UV light. After that, the same volume of absolute ethanol was added to the above-mixed solution, and ultrasonic treatment was performed for 5 min to uniformly disperse the yellow precipitate. Then, the mixture was centrifuged at 8000 rpm for 8 min, washed three times with anhydrous ethanol, and dried in a rotary evaporator at 50 °C for 1 h. The yellow powder obtained was the pure product of LA−Cu NCs@CS, which was stored at 4 °C for future use.

### 2.4. Construction of Fluorescent Probe Based on LA−Cu NCs@CS

First, an anhydrous ethanol solution of LA−Cu NCs@CS (2 mg mL^−1^, pH = 7.40) was prepared, and then a series of H_2_O_2_ aqueous solutions with different concentrations (0.8, 2, 8, 16, 32, 64 and 128 µM) were added to 1 mL of LA−Cu NCs@CS solution. After reacting at room temperature for 5 min, the fluorescence emission spectrum was measured with fluorescence spectrophotometry. As the concentration of H_2_O_2_ increased, the intensity of the fluorescence spectrum showed a gradually decreasing trend, a linear curve was drawn, and a linear regression equation was obtained by fitting, which was a model of the fluorescent probe.

In addition, under optimized conditions, different methodological verifications of the fluorescent probe were also carried out. Selectivity: Explore the interference of different cations and anions on the fluorescence of LA−Cu NCs@CS; Stability: the fluorescence intensity changes of LA−Cu NCs@CS within a certain time; Reproducibility: Three parallel experiments were performed to verify that the LA−Cu NCs@CS fluorescent probe has a good response to H_2_O_2_.

### 2.5. Detection of H_2_O_2_ Using LA−Cu NCs@CS

To investigate the performance of the fluorescent probe in actual detection, different actual water samples were analyzed. After filtering the tap water and lake water samples with a 0.22 µm filter membrane, the fluorescent probe was used to test the tap water and lake water samples to find no H_2_O_2_, and then the spiked recovery method was used for H_2_O_2_ determination. Three groups of H_2_O_2_ spiked solutions of different concentrations (0.8, 8, 32 µM) were added to the treated tap water and lake water samples, and the fluorescence emission spectrum was tested after incubating at room temperature for 5 min. Three experiments were performed in parallel, and the relative standard deviation (RSD) was calculated. The measured fluorescence intensity value was substituted into the constructed linear regression equation to calculate the final spiked recovery rate.

## 3. Results and Discussion

### 3.1. Morphology of LA−Cu NCs and LA−Cu NCs@CS

**Figure 1:**
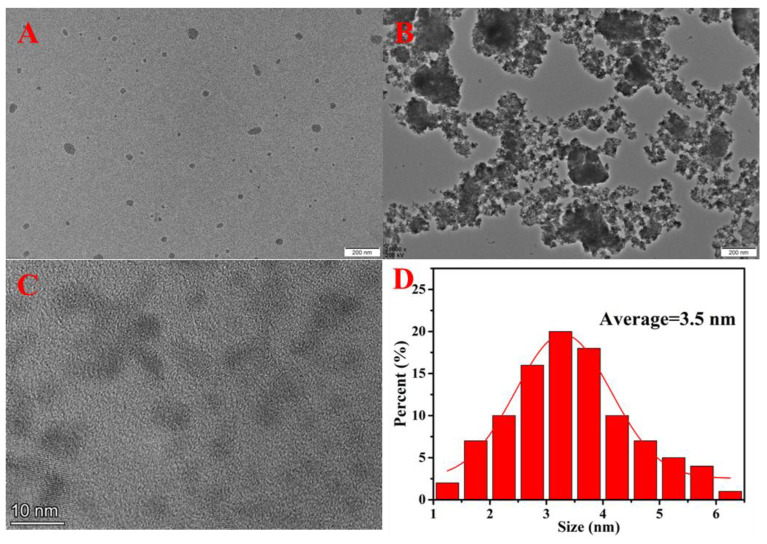
The TEM images of (**A**) LA−Cu NCs, (**B**) LA−Cu NCs@CS, (**C**) HRTEM image of LA−Cu NCs@CS, The diameter distributions histogram of (**D**) LA−Cu NCs@CS.

Figure 1 demonstrates the morphology and nanometer size of the prepared LA−Cu NCs and LA−Cu NCs@CS by TEM and HRTEM. LA−Cu NCs showed spherical dispersion in shape with an average size of 3.1 nm (Figure 1A and Figure S2). In the presence of CS, LA−Cu NCs appeared to aggregate in an aqueous solution, which was also confirmed by TEM. LA−Cu NCs@CS did show an aggregate state in Figure 1B. The HRTEM image (Figure 1C) showed that the single crystal lattice of LA−Cu NCs@CS was approximately 0.33 nm (3.3 Å), which corresponded to the 102 planes of Cu [30], and the average size was 3.5 nm (Figure 1D). The characterization of the morphology above showed that the successful combination of CS and LA−Cu NCs, the formation of an aggregated state, and the size distribution had not changed significantly.

### 3.2. Optical Performance Analysis

**Figure 2:**
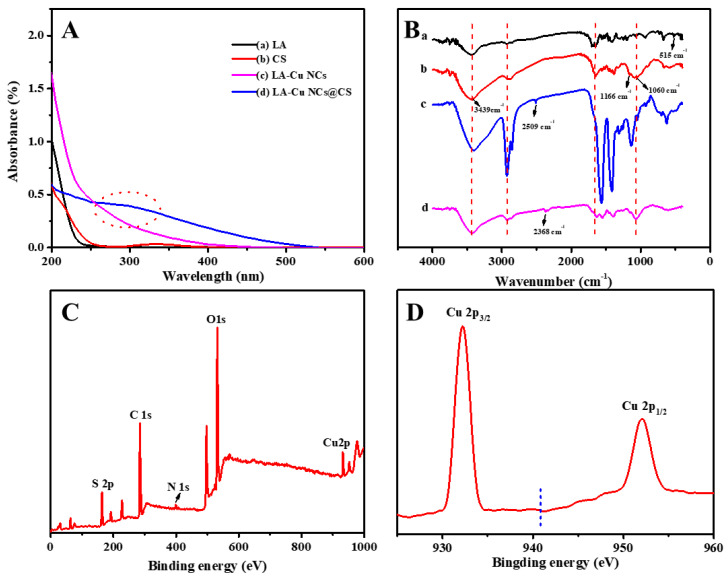
(**A**)The UV−vis and (**B**) FT−IR spectra of LA (a), CS (b) LA−Cu NCs (c) and LA−Cu NCs@CS (d), XPS spectra of (**C**) LA−Cu NCs@CS and (**D**) Cu 2p region.

As shown in Figure 2A, the UV-vis spectra showed that synthesized LA−Cu NCs had a broad absorption shoulder at 250~300 nm; LA−Cu NCs@CS had a strong and broad absorption peak at approximately 300 nm. The change in the spectrum proved that the encapsulation of the LA−Cu NCs by CS caused variation in the molecular structure of the light−absorbing group, which led to a shift in the absorption peak. In the FT−IR spectra (Figure 2B), the infrared peak at 515 cm^−1^ in the curve (a) was located in the disulfide bond (−S−S−) in LA. Under alkalinity and the reduction of NaBH_4_, the −S−S− in LA−Cu NCs was broken and bonded with the reduced Cu^2+^ to form a −S−Cu bond and the characteristic peak position was at 2509 cm^−1^ (curve c). The infrared peaks at 1060 cm^−1^, 1066 cm^−1^, and 3439 cm^−1^ in curve (b) were the C−O and C−O−C stretching vibration peaks and the characteristic peaks of amino (−NH_2_) in CS, respectively. When LA−Cu NCs were encapsulated by CS, the peak position of −S−Cu (2368 cm^−1^) was redshifted under the influence of the electron−donating group (−NH_2_, −OH) in CS (curve d), and the infrared peak positions of other groups were unchanged.

XPS was employed to perform chemical element and valence analysis. The XPS total spectrum showed that there were five peaks of C 1 s, N 1 s, O 1 s, S 2p, and Cu 2p under the corresponding binding energy, corresponding to the existence of C, N, O, S, and Cu elements in LA−Cu NCs@CS (Figure 2C), which was consistent with expectations. In the Cu 2p spectrum (Figure 2D), the characteristic peaks at 932.2 eV and 952.3 eV were attributed to Cu 2p3/2 and Cu 2p1/2 of Cu(0), respectively. In addition, there was no obvious absorption peak at 943.5 eV, which indicated that Cu(II) in LA−Cu NCs@CS was almost nonexistent and had been completely reduced. The difference in binding energy between Cu(0) and Cu(I) was only approximately 0.1 eV, indicating that the valence state of Cu in LA−Cu NCs@CS may be 0 or +1 [33]. In addition, the EDS spectrum clearly shows the inclusion of C, N, O, S, and Cu elements (Figure S1), which was consistent with the XPS spectrum test result, and the mapping element distribution of the characteristic elements of N, O, S, and Cu was also very uniform. In addition, EDS Mapping analysis of LA−Cu NCs@CS was performed to further verify the successful synthesis of the materials. As shown in Figure S1, the characteristic spectra of Cu, N, S and other elements can be seen from the figure, indicating that the elements in LA−Cu NCs@CS are closely combined.

**Figure 3:**
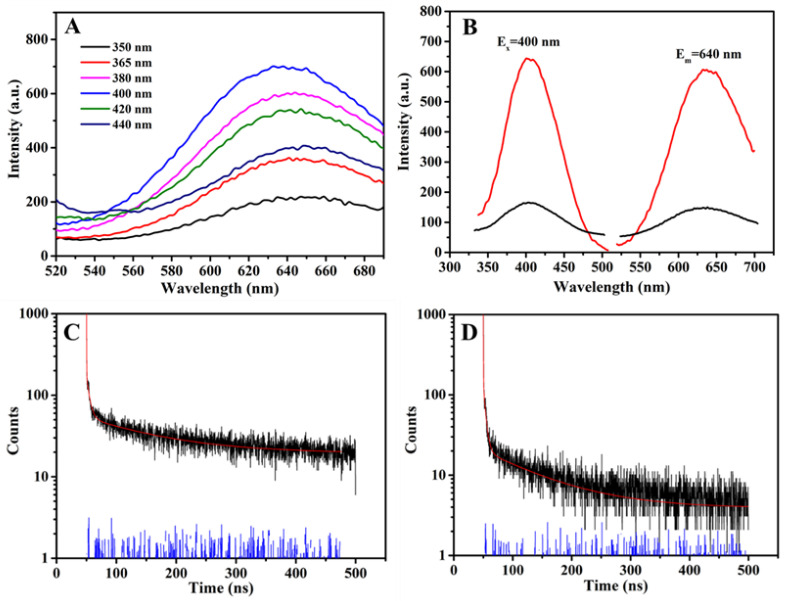
(**A**) Emission spectra of LA−Cu NCs@CS with different excitation wavelengths (**B**) Excitation and emission spectra of LA−Cu NCs (black line) and LA−Cu NCs@CS (red line). Time−resolved fluorescent decay spectra of (**C**) LA−Cu NCs and (**D**) LA−Cu NCs@CS.

As shown in Figure 3A, under different excitation wavelengths (350–440 nm), LA−Cu NCs@CS had the maximum fluorescence emission when the excitation wavelength was 400 nm. At the optimal excitation wavelength, the excitation and emission spectra of LA−Cu NCs and LA−Cu NCs@CS showed that the fluorescence emission intensity of LA−Cu NCs was relatively weak, and the fluorescence intensity became approximately four times that of the original after adding CS (Figure 3B). After CS encapsulated LA−Cu NCs to form LA−Cu NCs@CS, it triggered the aggregation of fluorophores and increased the fluorescence intensity. Figure 3C shows the fluorescence decay spectrum of LA−Cu NCs, whose fluorescence lifetime was 77.94 ns, as calculated by the weighted average method [34]. After adding CS, the fluorescence lifetime calculated by the same method was 58.03 ns (Figure 3D), which was less than that without CS. This result indicated that the presence of CS can enhance the fluorescence intensity of LA−Cu NCs.

### 3.3. Optimization of Conditions

**Figure 4:**
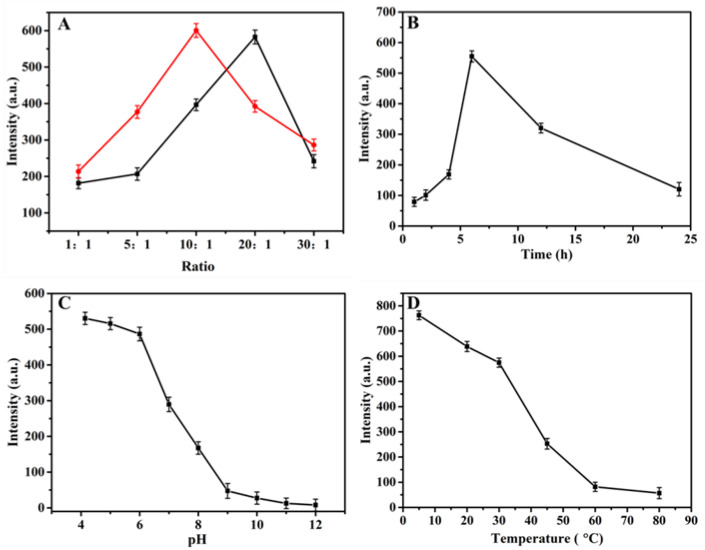
Optimization results of (**A**) the molar ratio of LA and Cu^2+^ (black line) and the ratio of LA−Cu NCs solution volume to CS (red line), (**B**) reaction time (25 °C), (**C**) pH value and (**D**) reaction temperature (reaction time: 6 h).

During the synthesis process, the effects of different reaction conditions on the fluorescence performance of LA−Cu NCs@CS were determined, including the ratio of raw materials, reaction time, pH, and reaction temperature. Figure 4A shows the fluorescence emission intensity of the different molar ratios of LA and Cu^2+^ (1:1, 5:1, 10:1, 20:1, and 30:1) and the volume ratios (1:1, 5:1, 10:1, 20:1, and 30:1) of the total volume of the LA−Cu NC solution under excitation at 400 nm. As the ratio of LA increased, the fluorescence intensity gradually increased, reaching a maximum of 20:1. At the same time, the fluorescence intensity was the largest when the ratio of the total volume of the LA−Cu NC solution to the volume of the CS solution was 10:1. The appropriate reaction time was not only conducive to the benign growth of LA−Cu NCs@CS molecules but could also avoid the excessive growth of the reaction time that would affect the fluorescence performance. The fluorescence emission intensity at different reaction times in Figure 4B shows that the fluorescence intensity was the maximum at 6 h, which was the time for the final reaction to prepare LA−Cu NCs@CS. The initial pH value of the synthesized LA−Cu NCs@CS was 4.14. When the pH was adjusted by adding 1 M NaOH, it was found that the fluorescence intensity changed little at pH 4–6, and the fluorescence intensity decreased significantly when the pH value was greater than 6 (Figure 4C), which indicated that the weakly acidic environment (pH = 4–6) was suitable for the synthesis of LA−Cu NCs@CS. The effect of reaction temperature on fluorescence was also further optimized. As shown in Figure 4D, as the reaction temperature increased, the fluorescence intensity decreased greatly, which demonstrated that low temperature was more suitable for the synthesis of LA−Cu NCs@CS, and the final preparation of LA−Cu NCs@CS was carried out in an ice bath environment (5–10 °C).

### 3.4. Fluorescence Response of LA−Cu NCs@CS in the Presence of H_2_O_2_

H_2_O_2_ is an important and abundant reactive oxygen species in organisms that play an important role in maintaining the physiological balance of cells in the body, and H_2_O_2_ has a wide range of applications in the food, environment, pharmaceutical, and textile industries. However, excessive discharge of sewage and waste liquid containing a large amount of H_2_O_2_ will cause harm to the environment and water resources. At present, the analysis and monitoring of H_2_O_2_ have always been the focus of related research. In this study, it is found that the presence of H_2_O_2_ will cause the fluorescence of the LA−Cu NCs@CS solution to be significantly reduced. Therefore, the construction of a probe based on LA−Cu NCs@CS will show good prospects in the detection of H_2_O_2_.

**Figure 5:**
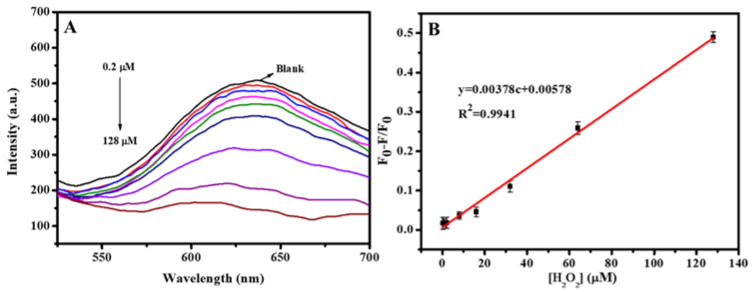
(**A**) The emission spectra of LA−Cu NCs@CS with increasing H_2_O_2_ concentration (**B**) Linear relationship between H_2_O_2_ concentration and LA−Cu NCs@CS FL quenching.

Figure 5A and Table S1 show the changes in fluorescence intensity after adding a series of concentrations (0.2, 0.8, 2, 8, 16, 32, 64, and 128 µM) of H_2_O_2_ to LA−Cu NCs@CS for 5 min at room temperature. With increasing H_2_O_2_ concentration, the fluorescence intensity of LA−Cu NCs@CS showed a declining trend, and the greater the H_2_O_2_ concentration, the higher the fluorescence quenching degree. Figure 5B shows the linear relationship between the H_2_O_2_ concentration and LA−Cu NCs@CS fluorescence intensity ratio ((F_0_ − F)/F_0_). Where F_0_ and F are the fluorescence intensities of the LA−Cu NCs@CS solution without H_2_O_2_ and with different concentrations of H_2_O_2_, respectively. There was a good linear relationship between LA−Cu NCs@CS and the fluorescence intensity ratio. The linear equation was (F_0_ − F)/F_0_ = 0.00378C + 0.00578 (R^2^ = 0.9941), where C is the H_2_O_2_ concentration. The LOD of the probe for the H_2_O_2_ response was 47 nM (S/N = 3). In addition, to reflect the good detection performance of this method for H_2_O_2_, the detection of H_2_O_2_ by different methods was compared with the methods mentioned in this study. As shown in Table 1, compared with other nanomaterials [35,36,37,38,39], this method had a lower LOD for H_2_O_2_ detection. The above results showed that the fluorescent probe based on LA−Cu NCs@CS had a good performance and could achieve sensitive detection of H_2_O_2_. Moreover, this result was obtained by using three different hydrogen peroxide probes on the same day, which also proved that the probe had little difference in the detection performance of hydrogen peroxide during the day with acceptable RSD (2.21%).

### 3.5. Stability and Selectivity

**Figure 6:**
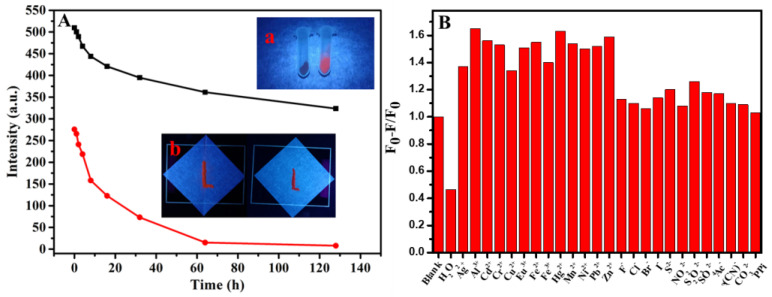
(**A**) The stability test of LA−Cu NCs (lower, red line) and LA−Cu NCs@CS (upper, black line), Insert: (a) Photos of the solid powder products of LA−Cu NCs (left) and LA−Cu NCs@CS (right) under UV light; (b) The photos of LA−Cu NCs@CS powder under UV light before (left) and after (right) two months. (**B**) Fluorescence responses of the fluorescence probe towards H_2_O_2_ and various metal ions.

Stability and selectivity experiments were performed separately to evaluate the applicability of the probe based on LA−Cu NCs@CS. Figure 6A shows the fluorescence intensity changes of LA−Cu NCs and LA−Cu NCs@CS prepared under the same conditions at different periods after storage at room temperature for approximately five days. In the first 20 h, the fluorescence intensity of LA−Cu NCs (red line) was reduced by approximately half, and the fluorescence intensity of LA−Cu NCs@CS (black line) decreased slowly within 20 h. Even after 128 h, the fluorescence intensity was also greater than the initial value of LA−Cu NCs. From this point of view, after adding CS to LA−Cu NCs, CS might have a certain stabilizing and protective effect on LA−Cu NC molecules, and the electron−donating groups (−NH_2_ and −OH) contained in CS were also beneficial to the fluorescence emission of LA−Cu NCs. It is worth noting that the difference in inner−day fluorescence intensity is slightly larger, and the relevant RSDs are between 3.77% and 4.84%. In addition, the red fluorescence intensity of the powder products of LA−Cu NCs@CS was much higher than that of LA−Cu NCs (inset a) under ultraviolet light. It is worth noting that the fluorescence intensity of the LA−Cu NCs@CS powder product under ultraviolet light hardly changed after storage for 2 months (inset b). Therefore, the addition of CS to LA−Cu NCs not only enhanced the fluorescence but also improved the stability, which provided a guarantee for practical applications.

Under optimized conditions, the interference of different metal ions and anions with the same concentration (32 µM) on the response of the LA−Cu NCs@CS system to H_2_O_2_ was explored separately. Figure 6B shows that only the fluorescence response of H_2_O_2_ had a significant change, and different metal ions and anions had almost no effect on the H_2_O_2_ probe test process, which indicated that the fluorescent probe based on LA−Cu NCs@CS had good anti-interference ability for H_2_O_2_ detection.

### 3.6. Actual Detection of H_2_O_2_

To verify the feasibility of the prepared fluorescent probe for H_2_O_2_ detection, we tested tap water and lake water samples and the presence of H_2_O_2_ was not detected. Then, the recovery rate was calculated using the spiked recovery method. First, the tap water and lake water were filtered with 0.22 µm membranes, and then the filtered tap water and lake water were used to prepare H_2_O_2_ with different concentrations (0.8, 8, and 32 µM) as the spiked samples. Finally, the linear equation of (F_0_–F)/F_0_ and H_2_O_2_ concentration was employed for the calculation of the final recovery rate. The spiked experiment was performed three times in parallel, and the results are shown in Table 2. The recovery rate of tap water was 95.88–98.44%, the recovery rate of lake water was 94.38–102.5%, and the total RSD ranged from 2.9% to 4.5%.

Accordingly, the fluorescent probe based on LA−Cu NCs@CS had good sensitivity and selectivity, which opened up a new path for the analysis and monitoring of H_2_O_2_ in actual environmental water samples.

## 4. Conclusions

In summary, we report a novel method that is used to construct a probe for detecting H_2_O_2_. The as-synthesized LA−Cu NCs@CS had good nanomorphology and superior luminescence properties. The linear range of the method was from 0.2 µM to 128 µM, and the limit of detection was 47 nM. In addition, this probe exhibits strong anti-interference ability in water samples with excellent selectivity and reproducibility. Furthermore, the synthesis of LA−Cu NCs@CS was simple and environmentally friendly, inexpensive, and served as a reference for the quantitative analysis of H_2_O_2_ in natural water and other water samples.

## Figures and Tables

**Table 1:** Comparison of different nanomaterials for H_2_O_2_ detection.

Nanomaterial	Linear Range/μM	Detection Limit/μM	Ref.
carbon quantum dots	0.5~50	0.2	[35]
gold nanoclusters capped by hemoglobin	0.5~700	0.21	[36]
Fe, N−incorporated carbon nanotubes	0.1~100	0.068	[37]
naphthalene backbone and a boric acid ester	1~250	0.7	[38]
sulfonated porphyrin	1~8	0.32	[39]
LA−Cu NCs@CS	0.2~128	0.047	This work

**Table 2:** Detection of H_2_O_2_ in actual water samples.(n = 3).

Samples	Added (µM)	Found (µM)	Recovery (%)	RSD (%)
Tap water	0	0	-	-
	0.80	0.767	95.88	3.6
	8.00	7.860	98.25	4.5
	32.0	31.50	98.44	3.7
Lake water	0	0	-	-
	0.80	0.755	94.38	2.9
	8.00	7.650	95.62	3.5
	32.0	32.80	102.5	3.4

## Data Availability

Not applicable.

## Supplementary Materials

The following supporting information can be downloaded at: https://www.mdpi.com/article/10.3390/bios13030361/s1, Figure S1: The EDS image of LA−CuNCs@CS; Figure S2: The diameter distribution histogram of LA−Cu NCs.; Table S1: The corresponding fluorescence intensity under different concentrations of H_2_O_2_ from Figure 5A. Equation (S1): The equation to calculate the LOD.
