# Supplementary material for: Preparation of a Red−Emitting, Chitosan−Stabilized Copper Nanocluster Composite and Its Application as a Hydrogen Peroxide Detection Probe in the Analysis of Water Samples
Source: Biosensors (Basel). 2023 Mar 9;13(3):361. doi: 10.3390/bios13030361 (PMC10046763; doi:10.3390/bios13030361)
Supplement: Supplementary file 1 [file biosensors-13-00361-s001.zip › biosensors-2209306-supplementary.pdf]

biosensors

MDPI

Supplementary Material

Preparation of a Red-Emitting, Chitosan-Stabilized Copper Nanocluster Composite and Its Application as a Hydrogen Peroxide Detection Probe in the Analysis of Water Samples

Jiaojiao Lu [1], Dawei Wang [1], Xin Li [1], Wei Guo [2,*], Chunyuan Tian [1], Feng Luan [1] and Xuming Zhuang [1,*]

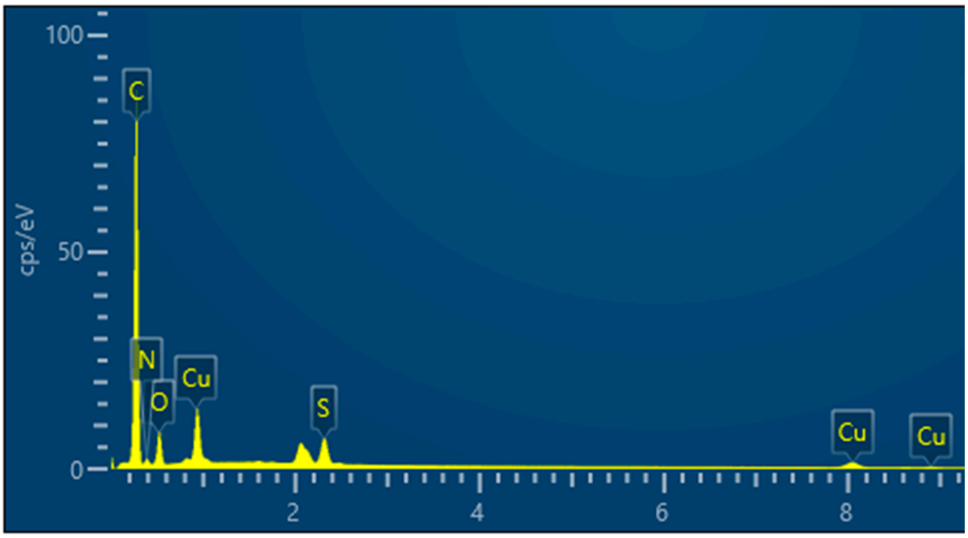

Figure S1. The EDS image of LA-CuNCs@CS.

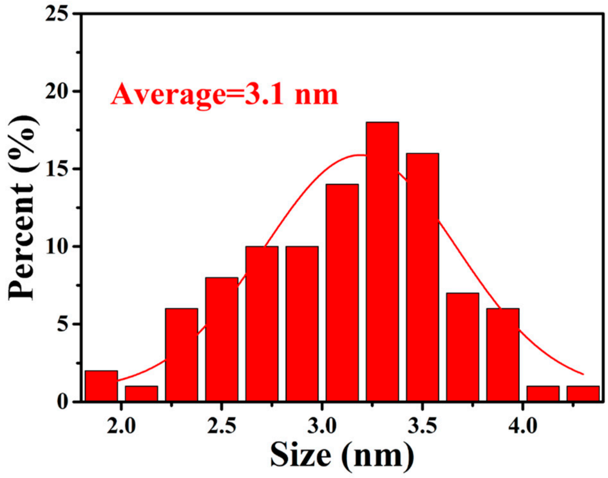

Figure S2. The diameter distribution histogram of LA-Cu NCs.

Table S1. The corresponding fluorescence intensity under different concentrations of H_2O_2 from Fig. 5A.

λ m/nm	640	640	640	640	640	640	640	640
**$[H_2O_2]$/μM**	0.2	0.8	2	8	16	32	64	128
**Intensity**	492.7	474.4	478.9	468.8	412.3	313	197.4	143.6

Equation S1. The equation to calculate the LOD is as follows:

$$LOD = 3S/N \tag{S1}$$

Where, N is noise and S is sensitivity of the method. The signal-to-noise ratio was obtained by analyzing the spectrum of the workstation.
